# Clinical practice guidelines for the diagnosis and treatment of patients with soft tissue sarcoma by the Spanish group for research in sarcomas (GEIS)

**DOI:** 10.1007/s00280-015-2809-5

**Published:** 2015-11-12

**Authors:** Xavier Garcia del Muro, Enrique de Alava, Vicenç Artigas, Silvia Bague, Alejandro Braña, Ricardo Cubedo, Josefina Cruz, Nuria Mulet-Margalef, Jose A. Narvaez, Oscar Martinez Tirado, Claudia Valverde, Ramona Verges, Joan Viñals, Javier Martin-Broto

**Affiliations:** Institut Català d’Oncologia L’Hospitalet, Barcelona, Spain; Hospital Universitario Virgen del Rocío, Sevilla, Spain; Hospital de la Santa Creu i Sant Pau, Barcelona, Spain; Hospital Central de Asturias, Oviedo, Spain; Hospital Puerta de Hierro Majadahonda, Madrid, Spain; Hospital Universitario de Canarias, Santa Cruz De Tenerife, Spain; Institut Català d’Oncologia L’Hospitalet, Barcelona, Spain; Hospital Universitario de Bellvitge, Barcelona, Spain; Instituto de Investigación Biomédica de Bellvitge (IDIBELL), Barcelona, Spain; Hospital Universitario Vall d’Hebron, Barcelona, Spain

**Keywords:** Soft tissue sarcoma, Clinical practice guidelines, Multidisciplinary management, Treatment

## Abstract

Soft tissue sarcomas (STS) constitute an uncommon and heterogeneous group of tumours, which require a complex and specialized multidisciplinary management. The diagnostic approach should include imaging studies and core needle biopsy performed prior to undertaking surgery. Wide excision is the mainstay of treatment for localized sarcoma, and associated preoperative or postoperative radiotherapy should be administered in high-risk patients. Adjuvant chemotherapy was associated with a modest improvement in survival in a meta-analysis and constitutes a standard option in selected patients with high-risk STS. In metastatic patients, surgery must be evaluated in selected cases. In the rest of patients, chemotherapy and, in some subtypes, targeted therapy often used in a sequential strategy constitutes the treatment of election. Despite important advances in the understanding of the pathophysiology of the disease, the advances achieved in therapeutic results may be deemed still insufficient. Moreover, due to the rarity and complexity of the disease, the results in clinical practice are not always optimal. For this reason, the Spanish Group for Research on Sarcoma (GEIS) has developed a multidisciplinary clinical practice guidelines document, with the aim of facilitating the diagnosis and treatment of these patients in Spain. In the document, each practical recommendation is accompanied by level of evidence and grade of recommendation on the basis of the available data.

## Introduction

Soft tissue sarcomas (STS) constitute an uncommon and heterogeneous group of tumours of mesenchymal cell origin, with an estimated incidence averaging five cases per 100,000 per year in Europe. STS can arise anywhere in the body, but most originate in the extremities, less frequently in the trunk, retroperitoneum, head and neck and viscera. They can occur at any age, and although more common in middle aged and older adults, they are also seen in children and young adults. Although STS comprise of more than 50 different histopathological subtypes, they share several clinicopathological features and are usually considered as a group for diagnostic and therapeutic purposes, with the exception of specific particularities of some subtypes such as rhabdomyosarcoma, gastrointestinal stromal tumour (GIST), extraskeletal osteosarcoma and Ewing sarcoma. STS require a multidisciplinary and complex therapeutic approach, involving pathologists, radiologists, surgeons, and radiation and medical oncologists, and should preferably be done in specialist centres.

In the recent years, there have been important advances in the understanding of the pathology and molecular biology of this disease. However, the advances in therapeutic results have been quite moderate. Moreover, due mainly to the rarity and complexity of the disease, the results in clinical practice are not always optimal. For this reason, the Spanish Group for Research on Sarcoma (GEIS) has updated its multidisciplinary clinical practice guidelines, with the aim of facilitating the diagnosis and treatment of these patients [1]. These guidelines have been developed by a multidisciplinary group of specialists in the different fields involved in sarcoma diagnosis and therapy. A bibliographic search of published articles was performed in the MEDLINE database (PubMed) and the Cochrane Library. Searches were limited to human studies, clinical trials, meta-analyses, clinical guidelines and consensus statements. Additionally, a review of abstracts of relevant, still to be published, phase III studies focused on STS therapy presented at relevant international oncology meetings like the American Society of Clinical Oncology (ASCO) meeting and European Society of Medical Oncology (ESMO) meeting, in the recent years, was performed. In a face-to-face consensus meeting, the different sections, written by different responsible experts, were presented to the entire group. The members then discussed the results and determined the level of evidence and the grade for each recommendation according to the ASCO guidelines [2]. The main objective of this document consists of providing clear practical recommendations about the different aspects involved in the management of this group of diseases, intended to help in the therapeutic decision-making processes and therefore contribute to improve STS patient’s care in Spain.

## Warning signs and indications for referral to specialist sarcoma centres

The symptoms of presentation of benign and malignant soft tissue tumours can frequently overlap, but there are clinical warning signs that may help to distinguish between both situations: tumour size >5 cm and recent increase in tumour size and depth location or pain. In almost three out of four of cases, patients who will be diagnosed with STS have at least one of the four warning signs. However, delays of several months from the first symptom/sign until the final diagnosis are not uncommon [3]. For this reason, any programme of early reference to expert centres must take into account these warning signs and should design efficient access from primary clinical centres to sarcoma specialized ones [4].

There is much evidence to support that early recognition and referral to a specialist center that provides a multidisciplinary diagnosis and therapeutic approach and treats a high number of cases annually, could improve outcome in patients with STS. A lot of data support that the first diagnostic approach is critical for functional results and disease control in patients with sarcoma. It is recommended that patients in which a STS is suspected should be referred to a specialized center before the biopsy is performed because complications and mistakes are more frequent in non specialized ones. The most frequent fault is the practice of excisional biopsies of deep tumours or superficial larger than 5 cm. There is a correlation between these procedures and the presence of affected surgical margins in the oncologic surgery (data from GEIS STS registry) [5]. The impact of affected surgical margins in primary surgery implies a poorer local control of the disease and also enhances the probability of amputation [6]. Regarding the definition of a sarcoma referral center, the most widely accepted is a center with a multidisciplinary specialist team who holds regular committee sessions, which is composed of surgeons, pathologists, radiologists, radiation and medical oncologists with wide experience in STS management [7]. However, there are no reliable data indicating the minimum number of cases per year that should be treated in a hospital to be considered as a reference center [7]. Several studies suggest better results in terms of local control, morbidity, and probably even in survival when patients are treated in specialized centres [7–9]. Therefore, centralized referral should preferably be done from the time of a sarcoma is suspected (III, B).

## Diagnostic approach to STS: imaging and pathology

### Local staging

Magnetic resonance imaging (MRI) is the modality of choice in the diagnosis and local staging of soft tissue sarcomas. MRI should be performed with intravenous contrast administration, and it is mandatory to obtain images in at least two planes. When MRI is contraindicated, contrast-enhanced multislice computed tomography (CT) should be performed, preferably with sagittal and coronal reconstructions. CT can also be appropriate as a modality of choice in retroperitoneal soft tissue sarcomas. MRI signs that suggest the diagnosis of sarcoma in a soft tissue mass includes: location deep to the fascia, size larger than 5 cm, and heterogeneity of signal intensity and contrast enhancement [10]. As there is some overlap in the MRI characteristics between soft tissue sarcomas and some benign soft tissue tumours, it is important to keep in mind that any lesion that cannot be unequivocally characterized by MRI as benign should be considered indeterminate, and requires biopsy.

In a patient presenting a soft tissue mass suggestive of sarcoma, the MRI report should provide the following information of the tumour:

- Size.
- Location (superficial or deep; compartment or extracompartmental).
- Anatomical limits.
- Relationship with neurovascular structures.
- Extension of perilesional oedema.
- Pattern of contrast enhancement.
- Suggestions of areas for biopsy.

The extent of perilesional oedema detected on MRI is important for treatment planning because the presence of viable tumour cells in this area has been demonstrated [11]. Local tumour staging should be performed prior to the biopsy to prevent bleeding and inflammatory changes caused by the biopsy that can distort the characteristics and extent of the tumour on MRI. It is recommended to obtain radiographs of the affected region to assess the existence of possible calcification within soft tissue tumour, evaluate possible bone erosion and exclude a bone tumour.

### Distant staging

Chest radiographs (frontal and lateral) and multislice chest CT are indicated in all cases of STS to rule out pulmonary metastases. Abdominal CT is indicated in the cases of myxoid liposarcoma of the extremities, given the high frequency of synchronous distant lesions in this histological subtype. The clinical benefit of performing an abdominal CT scan in cases of epithelioid sarcoma, synovial sarcoma, angiosarcoma and leiomyosarcoma should be assessed individually in each case. MRI study of the spine may be necessary in cases of myxoid liposarcoma due to its tendency to develop extrapulmonary metastases, and the limitations of bone scan or even positron emission tomography (PET) in the detection of spinal metastases in this histological subtype [12]. PET usefulness is limited to the detection of malignant transformation of neurofibromas in patients with neurofibromatosis type 1 [13], and in distant staging for cases of local recurrence of STS in which aggressive salvage surgery is considered.

### Biopsy

Percutaneous core needle biopsy (CNB), also known as “tru-cut biopsy”, is currently recommended for STS diagnosis [14], because it is minimally invasive and will not limit subsequent surgical interventions. Its accuracy is similar to that of the incisional biopsy, with the added benefit that it does not require hospitalization.

In the largest series of patients, CNB had a sensitivity of 99.4 % and a specificity of 98.7 % in the diagnosis of malignancy (sarcoma versus benign mesenchymal tumour) [14]. These percentages are very similar to those achieved with the incisional biopsy. In the same way, CNB may properly identify histological subtype and grade in 80 % of the cases [14]. It is recommended to perform the biopsy in the same hospital where the patient is treated, either by radiologists or surgeons trained in the practice of these procedures. Close cooperation with the pathologist is mandatory.

The use of radiological techniques to guide the CNB, mainly ultrasound and CT, has substantially improved diagnostic procedures. Image guidance allows more precise tumour location and helps to guide the biopsy to those areas of viable tumour. In selecting the areas for biopsy, it is essential to avoid cystic, necrotic or hemorrhagic tumour areas. Imaging studies should also be carefully evaluated to identify more aggressive areas (which usually show a higher degree of contrast enhancement) in order to define the tumour grade more accurately. The most commonly used CNB are between 14 and 18 G. In a recent review, the accuracy of this type of biopsy was not influenced by the gauge of the needle used [15]. In contrast, the number of biopsy passes (equal or above 4) and the length of the biopsy specimen obtained are factors that increase the diagnostic yield of CNB [15]. When planning a percutaneous biopsy, it is mandatory to avoid non-involved anatomical compartments, and it should be noted that the path of the biopsy and the scar should be resected by definite surgery. Hemostatic problems also must be corrected prior to the biopsy, due to the risk of tissue contamination caused by post-biopsy hematomas. STS are heterogeneous tumours, and a small sample obtained by this type of biopsy may underestimate the histological grade. Thus, unless a high degree appears in the percutaneous biopsy, definitive histological grade will be established in the surgical specimen. Due to the limitation on the size of the sample obtained by percutaneous biopsy, sometimes it is not possible to establish with certainty the histological diagnosis. In these situations, it may be necessary to perform incisional biopsy (III, B).

### Pathology and molecular pathology

Appropriate clinical information should be available for a correct interpretation of the initial biopsy. It is advisable in patients referred from other hospitals to ask for the biopsy material and/or surgical specimen for histological review and confirmation. In general, the core needle biopsy (tru-cut) is the diagnostic method of choice. Fine-needle aspiration cytology (FNA) is not recommended as the initial diagnostic procedure except in some centres with extensive experience. FNA is, however, suitable to confirm the presence of recurrence or metastasis of a sarcoma already known, and can be useful in some cases of round cell sarcoma, since molecular techniques may be performed on tumour imprints (touch preps).

Whenever possible and when it does not interfere with the diagnosis, it is recommended to freeze and preserve tumour fragments, and samples for cytogenetics (tumour imprints on slides pre-treated for immunohistochemistry), as well as liquid samples (plasma, serum) in biobanks. To avoid degradation of the tissue, the interval between biopsy and freezing should not exceed 30 min. The availability of a blood sample could add to the value of tumour tissues. Informed consent for biobanking should be obtained, enabling later analyses and research, as long as this is allowed by local and international rules.

#### Pathological diagnosis of soft tissue sarcomas

It is mainly based on morphology and immunohistochemistry. It must be in accordance with the 2013 World Health Organisation sarcoma classification [16]. This integrates morphological and immunohistochemical data, together with molecular cytogenetics. The histological tumour grade should be determined when possible; we recommend the system of the Fédération Nationale des Centres de Lutte Contre le Cance (FNCLCC), which is based on the evaluation of three histological parameters (tumour differentiation, mitotic index and percentage of necrosis), and defines three histological grades. In some sarcomas, histological type itself determines the aggressiveness of the tumour, and the histological grade does not provide additional prognostic information. Immunohistochemical study is useful to determine the type of tumour differentiation (muscle, neural, etc.), and, in turn, to rule out non-mesenchymal tumour types (carcinoma, melanoma, lymphoma…); but it does not provide information about the classification of the tumour as benign or malignant.

#### Indication of molecular studies

Use of molecular techniques is not necessary for the diagnosis of all cases of sarcomas. Molecular studies should be performed especially when:

- specific histological diagnosis is doubtful
- clinicopathologic presentation is unusual
- it may have prognostic/predictive relevance.

In the case of STS, the aim of the molecular study is to detect the presence of chromosomal translocations and gene fusions/gene rearrangements by the reverse-transcriptase polymerase chain reaction (RT-PCR) or fluorescence in situ hybridization (FISH).

#### Pathology report

Regarding the tru-cut (core needle) biopsy, in most cases it allows classification of the tumour (histology) and provides the degree of malignancy. The pathology report of a tru-cut biopsy should include histological type (when not possible it may be useful to try to classify in the categories of spindle cell, pleomorphic, myxoid, epithelioid or round cell), histological grade and the results of any additional tests that have been made (immunohistochemistry and/or molecular biology) [17]. One limitation of the core biopsy is that it can miss a small high-grade region in a heterogeneous tumour. Therefore, when preoperative treatment is an option, radiological imaging may be useful in addition to pathology, in providing the clinician with information that helps to estimate the tumour grade (i.e. necrosis).

The pathology report of a resected specimen of sarcoma should include, in addition to the histological type and tumour grade, tumour description (size, location, percentage of necrosis and hemorrhage and relationship to adjacent structures), status of resection margins, satellite nodules and lymph nodes. An appropriate sampling of the tumour should be made; it is generally recommended to select a section for each centimetre of tumour diameter. In very large tumours, 10–12 blocks can be enough. The sections on the margins should be at right angles. The report should also include the result of the immunohistochemical and molecular studies, if performed.

## Treatment of localized disease

### Surgery

Surgery is the mainstay of treatment in localized STS. Although they are a heterogeneous group of neoplasms, the main principles of surgery are common to them all. Surgical treatment should be performed by expert surgeons and should be based on consensus decisions taken on a multidisciplinary board.

Wide excision of the tumour is the base of surgical treatment of STS, which implies *en bloc* resection of all the neoplasm, the neocapsule (or reactive peritumoural tissue) and a variable layer of surrounding healthy tissue, called surgical margin. It is not possible to define the standard surgical margin, because it depends on several factors: tumour size, tumour location (intra or extracompartmental), the anatomical compartment implied, histological grade, proximity to neurovascular structures and the previous treatments performed.

Depending of the characteristics of surgical margin, surgery can be classified into:

- *Intralesional* only accepted as a diagnostic biopsy (incisional biopsy).
- *Marginal* includes peritumoural reactive tissue, but not healthy tissue margins.
- *Wide* includes an appropriate margin of healthy tissue free of tumour. However, the presence of the fascia, perineurium, periosteum and vascular adventitia free of tumour are considered as adequate margins.
- *Radical* includes all the anatomical compartment in which STS has grown.
- *Contaminated* a surgical procedure of STS should be considered contaminated if sarcomatous tissue remains exposed during surgery. In these cases, local spread of malignancy could occur.

*In bloc* surgery with wide or radical margin is the right treatment, but marginal surgery could also be accepted in some cases of low-grade STS with extracompartmental location (for example, atypical lipoma).

Enneking, Spanier and Goodman have contributed to the improvement of the knowledge and efficacy of surgical procedures in STS by defining the concept of anatomical compartments as a close unit where the tumour is confined, allowing surgeons to plan the surgery better from oncological point of view [18]. The following list defines compartmental and extracompartmental locations of STS (Table 1).

**Table 1:** Soft tissue sarcoma: compartmental and extracompartmental locations

Compartmental	Extracompartmental
Hand–foot radius	Midfoot and hindfoot
Rear leg	Popliteal space
Anterolateral leg	Scarpa triangle
Anterior thigh	Intrapelvic region
Medial thigh	Hand palm
Posterior thigh	Antecubital fossa
Gluteal region	Axilla
Anterior forearm	Periclavicular area
Posterior forearm	Paraspinal region
Periscapular	Head and neck

Surgery of STS must be carefully planned, taking into account the results of imaging and biopsy (STS subtype and grade). Unplanned surgery is unacceptable, and it is one of the main causes of local failure.

Surgical procedure can be performed through ischaemia technique, but pneumatic cuff should be far from the tumour, and sanguinea expression should be avoided. Elevation of extremity for 10 or 15 min will provide sufficient blood emptying. Regarding skin incision, it should always be performed on the direction of *longitudinal axis* of the member affected and should also include biopsy or aspiration cytology scar and previous drainage path. Once tumour excision is completed, the ischaemia system is removed, and two important technical aspects should be carried out: complete hemostasis (which reduces the risk of hematoma appearance and healing delays) and placement of suction drain system (following the longitudinal axis of the incision and as much closer to it as possible). Thorough washing of the tumour bed is mandatory to minimize infectious complications, mainly because these kinds of surgeries tend to be long and harmful for tissues. Inserting metal clips (titanium is preferred) before closing is recommended, in order to properly localize the surgical tumour bed. After that, closing of the defect can be performed trying to avoid empty spaces in the surgical bed, and a weak pressure bandage can also be placed to ensure a good tissue recovery. Moreover, surgical specimen has to be marked to help pathologist to identify surgical margins properly. However, if contamination of the surgical field occurs during surgery, some procedures have to be done: close the gap opened in the tumour by suture, change surgical instruments, expand surgical margins and report it because it will be taken account at the time of planning adjuvant treatments.

### Salvage surgery and amputation

Adequate surgery with extremity preservation is usually possible in the vast majority of STS. However, amputation or disarticulation should be planned for the following scenarios:

- When free margins cannot be ensured with conservative surgery and they are tried to be obtained with more radical surgery.
- When wide infiltration of neurovascular bundles exists.
- When properly functional reconstruction is not feasible or the result is, at least, the same as conservative surgery.

If after STS surgery margins are not free, this initial surgery is considered suboptimal and, as a consequence and if it is possible, re-surgery should be planned in order to expand margins.

Surgery for expanding margins must be more radical, and therefore, reconstruction procedures tend to be more complex [19–21].

If after an initial STS treatment a local recurrence occurs, salvage surgery can be performed. However, before planning it, it is important to properly evaluate the existence of a possible multicentric recurrence and/or metastatic disease.

In general terms, recurrence surgery is more complicated and more aggressive (and the resection must include drain path drainage of previous surgery). In this situation, the percentage of amputations increases and reconstruction procedures are more often needed [22].

### Radiotherapy

Adjuvant radiotherapy (RT) is offered in addition to conservative surgery to optimize local control. There are two prospective randomized trials, one using brachytherapy (BRT) ant the other postoperative external beam RT (EBRT), which demonstrated the local control advantage of adjuvant RT over surgery alone in sarcomas [23, 24]. The study used EBRT, randomized 141 patients (91 with high-grade tumours, 38/91 < 5 cm, 50 with low-grade tumours, 16/50 < 5 cm) to receive or not receive postoperative RT [23]. In this prospective randomized trial, adjuvant postoperative EBRT was shown to result in a statistically significant reduction in local recurrences in patients with either high-grade or low-grade extremity tumours, without significant differences in overall survival. The conclusions of this study are pertinent only to patients who undergo a satisfactory local excision with negative or minimal microscopically positive resection margins. A second study evaluated post-operative BRT, randomizing 164 patients to BRT or no further treatment [24]. The improvement in local control was limited to patients with high-grade lesions and was not associated with a significant reduction in distant metastasis or improvement in disease-specific survival.

Post-operative and pre-operative EBRT have also been compared with each other in a randomized manner. The National Cancer Institute of Canada (NCIC) trial compared 50 Gy in 25 fractions pre-operatively and 66 Gy in 33 fractions post-operatively [25]. They accrued 182 evaluable patients (31 low-grade STS, 151 high-grade STS) for which eligibility criteria was the need for combined radiotherapy and surgery, that is, if tumour could not be excised with a minimum of 2 cm of healthy tissue or an intact fascial plane. The primary endpoint was the presence or absence of a major wound complication. Of the pre-operatively irradiated patients, 35 % showed wound complications compared with 17 % in the post-operative RT group (*p* = .01). No difference was found between the two groups in local control, progression-free or overall survival, although the study was not powered to evaluate these end points. Interstitial BRT has been demonstrated to be an effective method of delivering adjuvant RT, within a considerably shorter treatment time than EBRT, and with potentially smaller treatment volumes [24]. However, its relatively limited use could be attributed to lack of effectiveness in low-grade histology, and it is a complex and labour-intensive technique.

Regarding some specific technical issues, preoperative RT is delivered in once-daily 1.8- to 2-Gy fractions to a total dose of 50–50.4 Gy. The entire dose is prescribed to a single planning target volume (PTV). In the case of postoperative RT, a portion of the dose is ordinarily applied to a larger volume encompassing the surgical bed with appropriately safe margins. A careful review of the surgical and pathology reports is essential to optimally define the target volume and the dose level to be administered. Commonly applied doses are 45–50.4 Gy in once-daily 1.8- to 2-Gy fractions. This is followed by a smaller volume boost to the tumour bed. Typically, doses of 14–18 Gy are applied, resulting in a total dose of 63–66 Gy. The guidelines to construct the RT volumes are available [26, 27].

When RT can be administered with minimal toxicity, its significant contribution to local sarcoma control following limited resection is of value. When the expected toxicity of RT is high and the risk of local recurrence is predicted to be low (based on surgical margins, tumour size), surgery without adjuvant RT may be the treatment of choice. Specific recommendations are: wide excision is followed by radiation therapy as standard treatment of deep and high-grade (G2–3) STS (II, B). Radiation therapy is added in selected cases in the case of high-grade, superficial lesions or in the case of low-grade, deep, >5 cm lesions. Radiation therapy is added in selected cases in the case of low-grade, superficial, >5 cm and low-grade, deep, <5 cm lesions (II, B). Radiotherapy may be carried out pre-operatively or post-operatively (I, A).

### Adjuvant chemotherapy

The use of adjuvant chemotherapy has been studied in several clinical trials because of the risk of metastasis which is close to 50 % in the group of patients with high-risk STS. These studies, however, often provide conflictive results. Variations in the chemotherapy schedules and the selection criteria used in the different studies could partially explain the disparity in the results. A recent meta-analysis included a total of 18 phase III trials comparing adjuvant chemotherapy versus observation in resected localized STS. The regimens used included doxorubicin or epirubicin, associated with ifosfamide in five trials. The results showed that chemotherapy was associated with a risk reduction in overall recurrence of 10 % and an improvement in overall survival of 6 %. Moreover, the results suggested that the association of anthracyclines and ifosfamide is associated with an improved benefit [28].

A previous meta-analysis, including 14 trials, showed a greater benefit in the group of patients with extremity sarcomas [29]. A recent trial focused on patients with high-risk STS (high-grade, deep location and >5 cm) located in the extremities and girdles. The chemotherapy schedule included high doses of epirubicin and ifosfamide. After a median follow-up of 5 years, overall survival was significantly higher for patients treated with adjuvant chemotherapy [30], although that difference decreased after a longer follow-up [31]. Another trial, performed by the European Organization for Research and Treatment of Cancer (EORTC) not included in the above-mentioned meta-analysis, involved patients with high- and intermediate-grade STS at any site. The adjuvant chemotherapy consisted of doxorubicin and ifosfamide versus placebo. No differences in survival between both arms were found [32]. Nevertheless, an update of the meta-analysis including this study showed a difference in overall survival favouring adjuvant chemotherapy with a hazard ratio of 0.86 (95 % CI 0.79–0.97) [32].

Consequently, data from meta-analysis indicate that adjuvant chemotherapy using anthracycline-based regimens provides a significant, although limited, improvement in relapse and survival in patients with high-risk STS. For this reason, it constitutes a standard option of treatment in selected patients (IA). Its administration should be only considered in those patients with high-grade, deep and >5 cm tumours, especially if they are located in the extremities (IIA). However, close observation without chemotherapy administration is also a standard option, given its limited benefit and the risk of associated toxicity (IA). The decision of whether to treat or not should be made in each individual case, after discussion with the patient of potential benefits and risks. If chemotherapy is administered, a regimen including doxorubicin and ifosfamide is recommended (IIA). For patients younger than 65 years, the Frustaci and col. regimen [31] is recommended (IIB). The standard recommendation consists of five cycles, but the results of a recent randomized trial of neoadjuvant chemotherapy suggest that, in a perioperative setting, a total of three cycles could be enough [33] (IIB). Adjuvant therapy is particularly not recommended for the low-grade sarcomas, those <5 cm, retroperitoneal and visceral sarcomas and subtypes not sensitive to chemotherapy (IIB). In the absence of further data, the EORTC 62931 trial suggests that delaying the administration of radiotherapy until completion of adjuvant chemotherapy is not associated with a worse local control [32] but has the potential advantage of not compromising the optimal chemotherapy administration (IIB).

### Neoadjuvant chemotherapy with or without radiotherapy and response assessment

The number of studies regarding neoadjuvant chemotherapy in STS is limited, and most of them are small series and phase II trials. A randomized phase II trial compared three cycles of neoadjuvant chemotherapy with doxorubicin and ifosfamide versus surgery alone in high-risk STS. The study did not demonstrate any benefit of neoadjuvant chemotherapy in terms of survival or in relapse-free survival [34]. Recently, a randomized non-inferiority phase III study compared three cycles of pre-operative chemotherapy with epirubicin and ifosfamide versus the same regimen plus two additional adjuvant cycles of treatment after surgery. The results showed that three cycles were not inferior to five cycles in terms of recurrence and survival [33]. Additionally, several non-comparative phase II trials showed the feasibility of concurrent neoadjuvant chemo-radiotherapy with different regimens and provided promising results [35]. This strategy, giving the demonstrated activity of preoperative radiation [25], is being widely studied at present.

Therefore, despite the current setting of shortage of evidence, some practical recommendations regarding neoadjuvant therapy in STS may be made. It could be considered as an option in those cases with large STS that are marginally resectable or require very aggressive surgery without assuring clean margins (III B). In those cases, probably the combination of pre-operative radiation and chemotherapy might have advantages over either modality alone (IVB). Additionally, potential benefits of neoadjuvant chemo-radiotherapy in patients with high-risk STS could be early treatment of micrometastases, shrinking the tumour, facilitating surgery and testing in vivo tumour chemosensitivity. Nevertheless, in this population neoadjuvant chemotherapy with or without radiotherapy remains investigational.

Concerning radiological and pathological response assessment to neoadjuvant therapy, some specific considerations should be addressed. MRI is the imaging modality of choice in the assessment of response in these patients. The assessments of the volume or the signal intensity of the tumour by MRI are not reliable criteria of response to neoadjuvant therapy since the tumour volume increase may be due to oedema and hemorrhage changes in cases of good response, whereas a reduction in tumour volume is usually not obtained even in cases with good response. Contrast-enhanced dynamic MRI is a technique that is being actively studied in the assessment of response to neoadjuvant therapy. However, at present its use remains experimental. On the other hand, pathological response to neoadjuvant therapy could be evaluated in the resected specimen. Percentage of necrosis of 95 % at least (similar to that used in bone sarcomas) is usually recommended, but data regarding this issue are controversial [36]. Nevertheless, decreased cellularity and fibrosis also should be taken into account together with necrosis.

### Role of isolated limb perfusion

Hyperthermic isolated limb perfusion (ILP) is a therapeutic approach that can be planned in STS when optimal conservative surgical procedure is not feasible, either due to tumour or patient comorbidity. It allows the administration of much higher doses of chemotherapy avoiding the systemic toxicity. ILP in hyperthermia is a technique in which the circulation of the limb where STS is located is cut off from the rest of the organism through an extracorporeal circuit and treated with biocytotoxic agents. The liquid administered is at a higher temperature than the rest of the body (39 °C), intensifying its effect. The administration of tumour necrosis factor alpha (TNF-α) and melphalan has improved the responses [37, 38]. Two recent reviews have analysed the results of several studies including 518 patients with STS treated with ILP. The overall response rate was 70 %, and the rate of patients in whom limb-sparing surgery was possible was also 70 %, although the number of local and systemic relapses was high. However, these reviews showed that an improved standardization of methodology in studies that evaluate ILP is needed [39, 40]. Therefore, hyperthermic ILP with TNF-α and melphalan constitutes an option. It may be considered in patients with limb STS where conservative surgery is not feasible, in order to carry out subsequent surgery preserving limb function (III, B) and patients with disseminated STS in whom local surgery is contraindicated, but local palliative control is necessary (III, B).

## Treatment of metastatic disease

With current appropriate management, local control of STS is achieved in around 80–90 % of patients. However, approximately half of all patients with high-grade tumours will develop metastatic disease and most of them will die from it.

### Surgery

Patients with exclusive pulmonary metastasis should be evaluated for surgery. The decision of metastasectomy should be based on disease-free period following primary surgery (ideally greater than one year) and total number of lesions (III, B). Complete resection of pulmonary metastases in the group of selected patients with these favourable prognostic features achieves up to 20 % long-term survival [41–45]. Prior restaging should be performed to rule out local recurrence or other sites of metastases. There is no clear evidence of benefit associated with the administration of adjuvant chemotherapy after resection of metastases in STS. In contrast, in patients with synchronous lung metastases, short disease-free interval or high number of lesions, chemotherapy should be the initial treatment. Subsequent surgery if benefit is achieved from chemotherapy, however, could be an option (IV, C).

### Chemotherapy and targeted agents

#### First-line treatment (doxorubicin and ifosfamide)

Doxorubicin and ifosfamide are the most active drugs and, given sequentially or in combination, constitute the standard treatment for advanced STS [46] (I, A). The association of doxorubicin and ifosfamide increased the response rate and toxicity but did not improve survival in randomized trials [47, 48] (I, A). High doses of ifosfamide (>10 g/m^2^) and doxorubicin regimens with GCSF support are also associated with increased response and toxicity but did not show improvement in survival in randomized trials [49, 50] (I, A). Therefore, the recommended first-line treatment is doxorubicin at 75 mg/m^2^. Epirubicin could also be an alternative to doxorubicin. Ifosfamide constitutes an alternative for contraindication of doxorubicin or a second-line treatment after doxorubicin failure. Ifosfamide in monotherapy is used at doses of 5–9 g/m^2^, although some data suggest that the higher doses are the most active. However, the use of a combination regime of both drugs could be justified when obtaining an objective response to improve symptoms or when performing surgery is important (III, B). Interestingly, some data suggest these could be some differences in the sensitivity to chemotherapy according to the different histological subtypes. For this reason, at present several studies are being performed on specific subtypes.

#### Second-line chemotherapy and beyond

Second-line therapy for advanced or metastatic unresectable disease is always palliative. Thus, close clinical observation may be an option for asymptomatic patients, especially for those with low-grade tumours or known low responsive entities. For symptomatic patients with poor performance status, radiotherapy or best supportive care alone is appropriate options. Symptomatic patients with good performance status are good candidates for clinical trials. Outside the context of clinical trials, conventional systemic therapy should be offered.

Trabectedin has been broadly explored in several phase I–II trials. Although the objective response rates were modest, a higher progression arrest rate was observed, especially in liposarcoma (notably myxoid liposarcoma, 88 %) [51], synovial sarcoma and leiomyosarcoma, but also in other tumour types. Tumour response had a particular pattern with early decrease in tumour density on CT or decrease in MRI contrast enhancement, followed by delayed tumour shrinkage. A randomized phase II trial in previously treated advanced liposarcoma or leiomyosarcoma comparing trabectedin infusion 1.5 mg/m^2^ over 24 h every 21 days versus a weekly scheme over 3 h demonstrated the superiority of the 24-h infusion in terms of time to progression [52]. Based in these studies, trabectedin was approved in Europe for patients with STS after progression to doxorubicin and ifosfamide or in patients ineligible for these treatments. The preliminary results of a recently reported phase III trial showed that trabectedin improves disease control in comparison with the classically standard second-line DTIC, in advanced pre-treated metastatic liposarcoma or leiomyosarcoma [96] (I, A). Premedication with dexametasone and administration through a central venous access device are recommended.

Gemcitabine has been evaluated in several phase II trials showing limited activity (II, B). If used, a fixed dose rate infusion (10 mg/m^2^/min) is usually recommended. DTIC alone has also limited activity, with 18 % short living responses [53]. However, the superiority of the combination of gemcitabine (1800 mg/m^2^ at 10 mg/m^2^/min) with DTIC (500 mg/m^2^) every 14 days versus DTIC alone has been reported in a randomized phase II trial in terms of median progression-free survival (PFS) and overall survival (OS), with a favourable toxicity profile [54]. The benefit of this combination appears to be more pronounced in leiomyosarcoma, although other subtypes may also benefit (II, B). Docetaxel in monotherapy is not active in STS, but in combination with gemcitabine has demonstrated interesting responses, especially in uterine leiomyosarcoma. A multicentric randomized phase II trial showed response rates of 16 versus 8 % and superior median PFS and median OS for the combination versus gemcitabine alone [55]. Patients with leiomyosarcoma and undifferentiated pleomorphic sarcoma appeared to be benefitted the most, but at the expense of greater toxicity. Contradictory results have been reported in another small phase II trial, particularly for non-uterine leiomyosarcomas [56] (II, C).

A randomized double-blind phase III study (the PALETTE trial) [57] comparing pazopanib (800 mg daily) versus placebo in 369 patients with non-adipocitic sarcomas progressing after first-line chemotherapy (including an anthracycline) showed a benefit in median PFS and disease stabilization favouring pazopanib (4.6 vs. 1.6 months, 67 vs. 38 %, respectively) with no significant difference in OS. All the included subtypes seemed to benefit to the same extent. Given the risk for serious hepatotoxicity, close monitoring of liver function tests is recommended, particularly in the first nine weeks of therapy (basal, week 3, 5, 7 and 9). Pazopanib constitutes an appropriate option after first line or thereafter progression in non-adipocitic sarcoma (I, A).

Additionally, a dose–response relationship has been shown for ifosfamide. Therefore, patients who have previously received ifosfamide may be treated with high-dose ifosfamide (>10 g/m^2^) [58] (III, B). Particular sensitivity has been reported for synovial sarcoma. Its main toxicities are hemorrhagic cystitis, renal and central nervous system toxicity. Concurrent administration of the uroprotectant mesna and appropriate hydration decreases the incidence of hemorrhagic cystitis.

For the majority of STS, there is no evidence that a particular drug sequence is better than another and probably most patients with good performance status benefit from being exposed to the largest number of available drugs (IV, C). As previously stated, some tumour types are specially sensitive to certain drugs, and this fact could help to select the second-line therapy, for example: high-dose ifosfamide for synovial sarcoma, trabectedin for myxoid liposarcoma and leiomyosarcoma, gemcitabine with docetaxel or with DTIC in leiomyosarcoma.

## Therapeutic considerations for specific STS subtypes

### Retroperitoneal sarcomas

Retroperitoneal sarcomas (RPS) are characterized by poor prognosis. More than half are high grade and adequate surgical margins are rarely obtained. The standard imaging procedure is a chest–abdominal CT scan. Fine-needle aspiration is not adequate for primary diagnosis. An extraperitoneal biopsy via core needle is the procedure of choice. Nevertheless, it is reasonable to avoid biopsy in the case of a resectable retroperitoneal mass with a clear-cut CT scan indicating adipocitic well-differentiated liposarcoma.

*En bloc* resection of the tumour including adjacent organs is the only curative treatment for RPS, negative margins being the main prognostic factor (IV B) [59]. Post-operative radiation is an option in highly selected patients with well-defined target regions (IV B) [60]. Adjuvant and neoadjuvant chemotherapy should not be routinely employed in RPS due to lack of evidence of benefit (IV B).

### Uterine sarcomas

Uterine sarcomas (US) are composed of different tumour entities: leiomyosarcomas, high-grade uterine sarcoma and sarcomas of endometrial stromal origin. Carcinosarcomas behave like epithelial carcinomas and are not covered by the following guidelines. Standard surgery of localized US consists of total abdominal hysterectomy plus double oophorectomy and full abdominal cavity exploration. It is not clear whether bilateral oophorectomy is always needed, particularly in low-grade US. Lymphadenectomy is not indicated. If an unsuspected US is diagnosed after surgery, a second-look intervention is not recommended if a total hysterectomy has been performed and macroscopic tumour does not remain. Adjuvant radiotherapy is controversial. Most available clinical trials are not optimally designed but tend to show a decrease in local relapse risk. No trial has proven an overall survival advantage [61]. Thus, adjuvant radiotherapy it is not routinely considered, but it is justifiable in selected cases with a high relapse risk (II, C). A clinical trial comprising a small number of patients with uterine leiomyosarcoma showed a benefit of adjuvant gemcitabine plus docetaxel compared with historical controls [62]. There is not enough evidence to recommend adjuvant chemotherapy, but it could be individually planned in some patients (III B).

Hormonal therapy with megestrol acetate, gonadotropin-releasing hormone (GnRH) analogues and aromatase inhibitors can delay progression for long periods of time in low-grade oestrogen receptor-positive endometrial stromal sarcoma, and it is preferred over chemotherapy as front-line palliative treatment (IV, C). Doxorubicin, ifosfamide, gemcitabine, taxanes, and trabectedin are active against US. We recommend a cautious approach favouring less toxic monotherapy options in the first place (IV, B). Positive results have been published for leiomyosarcoma patients treated with gemcitabine plus docetaxel as first- or second-line treatment [63]. It is acceptable to select either doxorubicin or this regime as first-line palliative chemotherapy (III, B).

### Desmoid tumours

Desmoid tumours (DT) represent a mesenchymal neoplasm of intermediate behaviour. They rarely metastasize, but show a marked tendency to local relapse with progressive increasing aggressivity. Surgery remains the mainstay of DT curative treatment. It is usually straightforward in the case of limb and chest-wall tumours, but can be much more challenging in abdominal disease. The aim of surgery is the macroscopic removal of the whole tumour while minimizing morbidity. Wide margins, even microscopically negative ones, do not justify on their own mutilating surgeries or functional sequels, as the prognosis of macroscopically resected (R1) patients do not depend on the microscopical status of the margins [64] (III, A). A watch-and-wait approach is also acceptable in the case of small, non-growing, asymptomatic extraabdominal tumours that do not impose a significant risk for nearby critical organs or structures. Non-treatment decisions should not be taken in the absence of a diagnostic biopsy (IV, B).

Radiotherapy is able to control even bulky disease for long periods of time [65]. It is recommended in several scenarios like unresectable tumours, R2 surgery, patients refusing surgical treatment, serious comorbidity that makes surgery too risky or cases in which radical surgery would impose serious functional or cosmetic sequels (III, B). Systemic treatment is appropriate for unresectable tumours, Gardner-related cases with multiple recurring DT, progressions in areas previously irradiated and functionally or aesthetically unacceptable surgery. Evidence-based options include non-steroidal anti-inflammatory drugs like sulindac (IV, D), anti-oestrogens (tamoxifen and toremifene) (IV, D), chemotherapy (low-dose methotrexate plus vinblastine or vinorelbine and liposomal doxorubicin) (III, B) and imatinib [66] (III, B).

### Angiosarcoma

Angiosarcoma (AS) is a heterogeneous type of sarcoma due to its age of presentation and location. Sometimes it is multifocal and can be associated to anaemia and coagulopathy [67–69] ]. The treatment of choice of localized AS is complete excision with wide margins. Adjuvant radiotherapy is recommended if optimal surgery is not feasible due to multifocality or difficulty in defining the true margins [67, 68] (IV, B). In advanced AS, systemic chemotherapy with either anthracyclines or taxanes are acceptable treatment options (II B). However, in the AS of the scalp, frequently seen in elderly patients, weekly paclitaxel is the drug of choice because it seems to have better response rate than anthracyclines [70–72]. Anti-angiogenic drugs like bevacizumab and sorafenib have also been tested in metastatic AS with promising activity [73, 74] (III, B).

### Alveolar soft part sarcoma

Alveolar soft part sarcoma (ASPS) often occurs in adolescents and young adults. Complete excision with wide margins is the treatment of choice. The administration of adjuvant radiotherapy follows the common guidelines for STS (IV, B). ASPS is not particularly sensitive to classic chemotherapeutic agents used in STS. However, ASPS has an upregulation of angiogenesis elements, and cediranib has proven to be active in advanced disease [75]. Several partial responses to sunitinib and bevacizumab have also been reported [76, 77] (IV, B).

### Dermatofibrosarcoma protuberans

Dermatofibrosarcoma protuberans (DFSP) is a cutaneous mesenchymal tumour of intermediate behaviour. Although DFSP metastasize exceptionally, it has an important local infiltrative ability. In cases of localized DFSP complete resection with wide margins (2–4 cm) is the treatment of choice. Mohs surgery can be planned to avoid major cosmetic defects (III B). Adjuvant radiation therapy should be considered when margins are positive and re-resection is not feasible [78–80] (IV, B). In unresectable, recurrent or metastatic DFSP, imatinib is recommended (III B) [81].

### Clear cell sarcoma

Clear cell sarcoma (CSS) tends to metastasize to lymph nodes, unlike other STS. In localized CCS, wide surgical resection is the mainstay of treatment. Elective lymphadenectomy or sentinel node biopsy could be also considered (IV C). Lymphadenectomy should be performed in cases of lymph node metastasis (IV B). The administration of adjuvant radiotherapy follows the common guidelines for STS (IV, B). In advanced CSS, response rate to chemotherapy is generally low [82–84]. However, some isolated responses have been described with antiangiogenic agents such as sorafenib or sunitinib [85, 86] (V, D).

### Solitary fibrous tumour

Solitary fibrous tumour (SFT) can be found in different locations, one of the most frequent is the pleura. Despite the majority of cases being benign, up to 40 % can be classified as malignant, which have a higher rate of local and distant recurrences [87, 88] (IV, B). The backbone of treatment of localized SFT is surgical resection with wide margins. Adjuvant radiation therapy can be administered (IV, B). In metastatic or locally advanced SFT, chemotherapy following the common guidelines for STS could be administered (III B). Moreover, antiangiogenic agents such as sunitinib or the combination of temozolomide plus bevacizumab constitute active options [89–91] (IV, B).

### Inflammatory myofibroblastic tumour

Clinical manifestations of inflammatory myofibroblastic tumour (IMT) are secondary to local tumour. However, some patients can present systemic symptoms like fever and weight loss, as well as laboratory abnormalities like anaemia, thrombocytosis and polyclonal hypergammaglobulinemia [92]. IMT is associated with rearrangements of the ALK (anaplastic lymphoma kinase) locus on chromosome 2p23.13 [93]. In localized IMT the mainstay of treatment is wide resection. Generally, the surgery is curative and adjuvant chemotherapy is not recommended. In advanced IMT ALK-translocated, ALK-inhibitors, like crizotinib, produce sustained responses and are recommended [93] (IV, B). Chemotherapy with different regimens has been classically used with variable efficacy (methotrexate plus vinorelbine, vincristine plus etoposide, cisplatin/carboplatin or ifosfamide regimens) [94, 95] and could constitute a second-line option (IV B).
